# NUMB alternative splicing and isoform-specific functions in development and disease

**DOI:** 10.1016/j.jbc.2025.108215

**Published:** 2025-01-23

**Authors:** Sascha E. Dho, Kamal Othman, Yangjing Zhang, C. Jane McGlade

**Affiliations:** 1 The Arthur and Sonia Labatt Brain Tumour Research Centre, The Hospital for Sick Children, Toronto, Ontario, Canada; 2 Department of Medical Biophysics, University of Toronto, Toronto, Ontario, Canada

**Keywords:** NUMB, protein isoforms, alternative splicing, cancer, development, PTB domain, splicing factor, endocytosis, trafficking

## Abstract

The NUMB gene encodes a conserved adaptor protein with roles in asymmetric cell division and cell fate determination. First described as an inhibitor of Notch signaling, multifunctional NUMB proteins regulate multiple cellular pathways through protein complexes with ubiquitin ligases, polarity proteins and the endocytic machinery. The vertebrate NUMB protein isoforms were identified over 2 decades ago, yet the majority of functional studies exploring NUMB function in endocytosis, cell migration and adhesion, development and disease have largely neglected the potential for distinct isoform activity in design and interpretation. In this review we consolidate the literature that has directly addressed individual NUMB isoform functions, as well as interpret other functional studies through the lens of the specific isoforms that were utilized. We also summarize the emerging literature on the mechanisms that regulate alternative splicing of NUMB, and how this is subverted in disease. Finally, the importance of relative NUMB isoform expression as a determinant of activity and considerations for future studies of NUMB isoforms as unique proteins with distinct functions are discussed.

## Evolution of NUMB isoforms: from *Drosophila* to human

**Figure 1:**
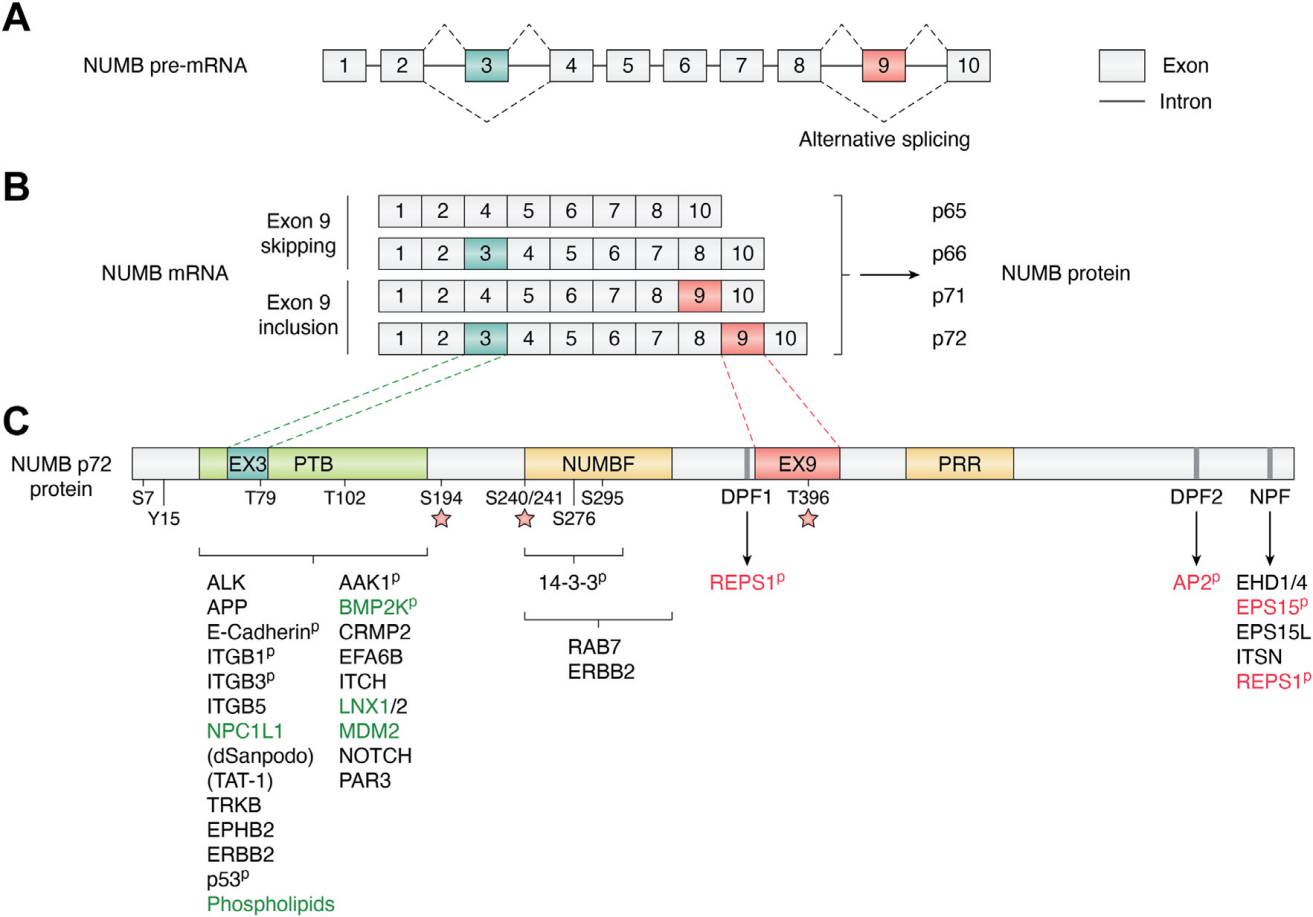
**NUMB alternative splicing.** A schematic representation of the splicing of NUMB pre-RNA (*A*) to form the major NUMB mRNA splice variants which are translated into a protein with molecular weights of 65, 66, 71, and 72 KDa. Exon 9 skipped (Ex9sk) NUMB protein isoforms are referred to in the text as p65, p66, and exon 9 included isoforms (Ex9in) as p71 and p72 as indicated (*B*). Exon numbering used in this review is based on the mouse NUMB transcript which has 10 coding exons (ENSEMBLE transcript: ENSMUST00000129335.8). The human NUMB transcript has 13 exons: 1 to 3 are non-coding and 4 to 13 are coding (ENSEMBLE transcript: ENST00000555238.6). The primary alternatively spliced exons are Exon 3 (Exon 6 in humans) and Exon 9 (Exon 12 in humans). *C*, NUMB p72 protein showing PTB domain, endocytic protein binding motifs, and localization of exon 3 and exon 9 encoded amino acids. The NUMB-interacting proteins and phosphorylation sites discussed in the text are shown below the protein schematic. Where known, differential binding is indicated as follows: exon 3 (*green*), exon 9 (*red*); (p) phosphorylation-dependent binding; red star indicates sites that exhibit greater phosphorylation in NUMB p72 compared to p66.

NUMB is a ubiquitously expressed adaptor protein that regulates multiple pathways, including Notch, Hedgehog, and MDM2/p53 signaling, endolysosomal trafficking, migration, calcium homeostasis, and DNA repair (1). Misregulated expression of NUMB is linked to a number of disease states including cancer where it acts as both a tumor suppressor and a tumor promotor. NUMB was originally identified in *Drosophila* (dNumb) as essential to cell fate determination and neuronal development (2). Through its ability to asymmetrically localize in dividing neuroblasts and sensory organ precursor cells, dNumb is differentially segregated into the daughter cells where it influences cell fate through its inhibitory effect on the Notch pathway (for a comprehensive review of this early work see (3, 4). NUMB first appeared evolutionarily in Metazoa while its paralogue Numblike (NUMBL) emerged in vertebrates (5, 6). They exist as single genes, with similar intron/exon arrangements. Both are characterized by the presence of an amino-terminal Phosphotyrosine Binding (PTB) domain and adjacent region conserved in NUMB family members (NUMBF), whereas the sequences C-terminal to NUMBF have little similarity aside from the presence of endocytic protein binding motifs as well as short proline-rich regions (Fig. 1). In invertebrates such as *Drosophila* and *Caenorhabditis elegans* two developmentally regulated NUMB isoforms are expressed from alternative translation initiation sites, a maternal form and a longer zygotic form (7, 8). However, during the evolution of the chordate lineage, multiple alternatively spliced NUMB mRNA transcripts emerged (6). Nine NUMB isoform transcripts have been described in *Homo sapiens* (9, 10, 11). This review will focus on the four most widely expressed and abundant isoforms that occur through alternative splicing of coding exon 3 (Ex3in: exon 3 included; Ex3sk: exon 3 skipped) and exon 9 (Ex9in: exon 9 included; Ex9sk: exon 9 skipped). Similar exons do not occur in dNumb nor NUMBL. The resulting proteins with molecular weights of 72 (Ex3in, Ex9in), 66 (Ex3in, Ex9sk), 71 (Ex3sk, Ex9in), and 65 (Ex3sk, Ex9sk) kDa (Fig. 1) are referred to as NUMB-1 to NUMB-4 respectively in the Uniprot database. However, for clarity, in this review specific protein isoforms will be referred to according to their molecular weights (p72, p66, p71, p65), while specific exons will be referred to as included (in) or skipped (sk).

Alternative splicing is an important mechanism for expanding the repertoire of gene function through proteome diversity. Regulated alternative splicing, for example, can result in the inclusion or exclusion of exons encoding short linear interaction motifs (SLiMs) and, to a lesser extent, modular domains that alter protein function (12, 13). Alternative splicing of NUMB Exon 3 and Exon 9 results in the insertion of 11 amino acids within the modular PTB domain, and 48 amino acids within the C-terminal region respectively (Fig. 1). It this review we describe what is currently known about the structure, function and expression of NUMB, with a focus on studies that have specifically addressed the role of particular isoforms. Many of the early studies of NUMB function did not address distinct functions of the protein isoforms that have come to light more recently. Therefore, this previous work has been reviewed and interpreted with a focus on the isoforms that were analyzed where possible. We describe the primary structural features of NUMB and how alternative splicing may contribute to functional differences between the major NUMB isoforms, including the role of NUMB isoforms in endo-lysosomal trafficking. Finally, the developmental regulation of NUMB alternative splicing will be described together with how this may be subverted in disease.

## Molecular structure and function of NUMB protein isoforms

### Inclusion of exon 3 in the PTB domain regulates protein-protein interactions

PTB domains are conserved modular domains which bind both proteins and acidic phospholipids. Based on the analysis of structure/function relationships they have been divided into three groups: SHC-like, IRS-like, and DAB-like (14). NUMB possesses a DAB-like PTB domain. The canonical peptide motif recognized by DAB-like PTB domains is FxNPxY, which adopts a type I β turn that forms a pseudo β sheet with the β5 strand and C-terminal alpha helix of the PTB domain. While SHC-like and IRS-like PTB domain recognition of this motif requires phosphorylation of the tyrosine residue, no such dependency exists with DAB-like PTB domains, including NUMB (15). Indeed, many PTB domains of this subtype exhibit binding to peptides with phenylalanine in place of the tyrosine residue (16, 17).

**Figure 2:**
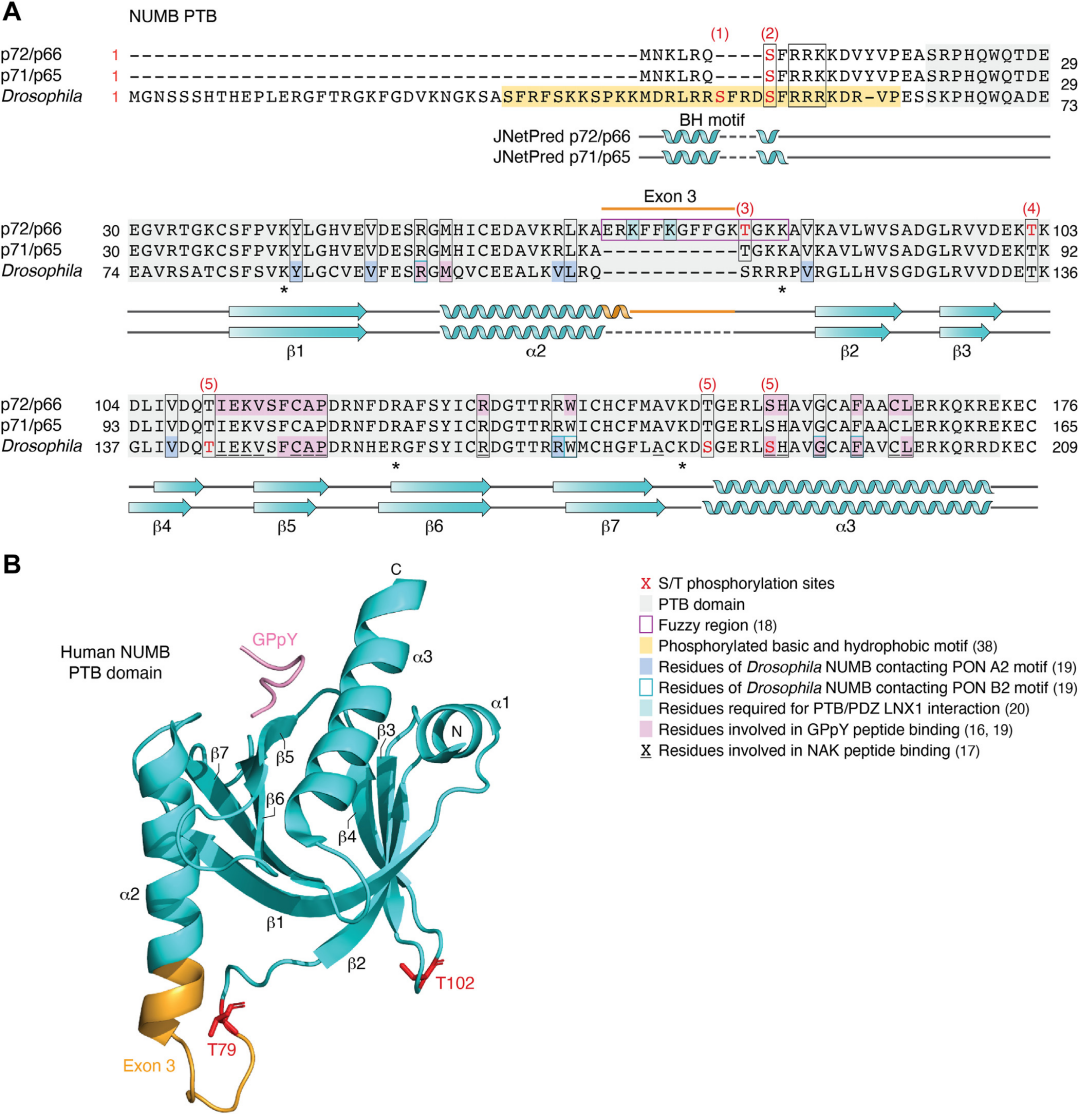
**Structure of NUMB PTB domain exon 3 splice variants.***A*, alignment of human NUMB PTB exon 3 splice variants with *Drosophila* NUMB. PTB domains are highlighted in *light grey* and amino acids shown by structural studies or mutational analysis to be important for function are indicated as shown in the legend. Stars (∗) indicate the amino acids conserved between NUMB and DAB1 PTB domain which were identified in the latter to be involved in phospholipid binding (Stolt *et al.* 2003 (30)). The Serine/Threonine phosphorylation sites shown are conserved between human and *Drosophila*; *red* amino acids indicate the protein in which the site was identified and are numbered according to the original citations: (1) Bailey and Prehoda 2015 (38); (2) Krieger 2015 (49), Bailey and Prehoda 2015 (38), Nishimura and Kaibuchi 2007 (43), Sato 2011 (46); (3) Krieger 2015 (49); (4) Sorensen 2008 (45); and (5) Ouyang 2011 (177). The predicted secondary structure is shown below the sequences; teal arrows indicate beta sheets and coils represent the alpha helices, and orange indicates exon 3 coded structure. The amino acid sequences of NUMB p72/p66 (NP_001005743.1/1–176), p71/p65 (NP_003735.3/1–165), and the zygotic A form of *Drosophila* NUMB (sp|P16554.2|NUMB_DROME/1–209) were imported in Jalview and aligned with Clustal Omega using the default settings. The Predicted secondary structure (JNETpred) was determined using the Jalview Web Service > JNet Secondary Structure Prediction Tool. Additional figure preparation was carried out using Affinity Designer graphics software and BioRender. *B*, visual representation of the exon 3 included PTB domain of human NUMB in complex with GPpY peptide. A model of the crystal structure of the exon 3 included PTB domain (*cyan*) of human NUMB (PDB: 5NJJ), visualized *via* PyMOL with exon 3 (*orange*), threonine residues 79 and 102 (*red*), and the PTB bound peptide GPpY (*pink*).

The structure of *Drosophila* NUMB PTB has been solved in complex with peptide ligands derived from known binding partners, NAK (NUMB Associated Kinase) and PON (Partner of NUMB), as well as an artificial peptide ligand, GPpY (Fig. 2*A* shows the peptide-interacting residues of dPTB domain conserved in mammalian NUMB) (15, 17, 18, 19). The structure of mammalian NUMB PTB domains with and without exon 3 have also been solved in complex with the GPpY peptide (18) (Fig. 2*B*). Comparison of the structure of the Ex3in PTB domain with Ex3sk PTB showed that the two align structurally and that the 11-amino acid exon 3 region forms a short extension of the alpha 2 helix followed by a flexible loop of positively charged residues bounded by additional positive residues within the core PTB fold and distinct from the canonical peptide binding surface (18) (Fig. 2, *A* and *B*). These structural analyses revealed that the NUMB PTB domain includes at least two independent surfaces for interaction with peptides (20, 21). Importantly, the presence of the 11 amino acid exon 3 sequence creates a surface for non-canonical peptide binding, and isoform-dependent modulation of the protein-protein interactions between NUMB and two E3 Ubiquitin Ligases, LNX1 and MDM2 (18, 20).

Both LNX1 and MDM2 exhibit much higher affinity binding to Ex3in PTB compared to Ex3sk PTB. LNX1, a multi-PDZ containing RING protein, was originally identified in a Yeast 2 Hybrid screen using Ex3in PTB (22). Structure-function analysis showed that the Ex3in PTB domain bound to LNX1 *via* a canonical PTB-binding motif (LDNPAY) but also through interactions with the LNX PDZ1 domain. Subsequent studies confirmed that the presence of exon 3 was necessary not only for LNX1 binding but also for *in vitro* ubiquitination of the NUMB N-terminus/PTB region, and *in vivo* ubiquitination and degradation of full length p72 and p66 (Ex3in) but not p71 and p65 (Ex3sk) proteins (20, 23). Mutation of positively charged lysines (K70A, K73A, and K78A; Fig. 2*B*) within the exon 3 encoded sequence abrogated LNX binding, suggesting the involvement of complimentary polar interactions. Similarly, multivalent interactions of the *Drosophila* NUMB PTB domain with type A and type B motifs in its binding partner PON have been demonstrated. While type A motifs resemble the fold of canonical NPxY motifs, B-motif binding is mediated by hydrophobic and polar interactions with a distinct PTB surface (Fig. 2*B*). This multi-valent interaction of dNUMB PTB and PON contributes to phase separation and basal cortex localization of the PON-NUMB complex (19). These observations suggest that multi-valent interactions are a conserved feature of the NUMB PTB domain that is regulated in vertebrates by alternative splicing of exon 3.

An exon 3-specific binding mechanism between Ex3in PTB and the acidic domain of the E3 ubiquitin ligase, MDM2 (amino acids 216–302) has also been defined (18). MDM2 inhibits the activity of the tumor suppressor p53 *via* ubiquitin mediated degradation. NUMB is known to stabilize p53 by binding MDM2 and preventing its interaction with p53 (24). Colaluca *et al.*, demonstrated that the PTB domain binding to MDM2 requires exon 3 encoded amino acids (Fig. 2*A*) but not the canonical PTB domain peptide binding pocket (18). Structural analyses showed that the exon 3 encoded sequence is intrinsically disordered with characteristics of a fuzzy region which makes hydrophobic and polar contacts in a flexible interface with the acidic domain of MDM2 (for more information about fuzzy complexes see review (25). Exon 3 sequences therefore also determine unique PTB domain binding specificity for targets such as MDM2.

### Exon 3 inclusion regulates PTB domain binding to lipids and enhances plasma membrane localization

In addition to peptides, PTB domains also bind acidic phospholipids (26) which in the context of proteins such as Dab1 and NUMB function to enhance association with the plasma membrane (14, 27, 28, 29). The lipid-binding region is composed of a cluster of basic residues (primarily arginine and lysine) located at the surface of the PTB domain tertiary structure. Protein structure analyses of DAB1 and DAB2 PTB domains, which are within the same subgroup as NUMB, show that this phospholipid binding region is located distant to that which binds peptide such that both lipid and peptide can bind simultaneously and independently (30, 31). The basic residues involved in DAB1 and DAB2 PTB domain phospholipid binding are conserved in NUMB (Fig. 2*A*). Whereas both Ex3in and Ex3sk NUMB PTB domains have been shown to bind phospholipids including PI3P, PI45P_2_ and PI3,4,5P_3_, the Ex3in PTB domain bound more strongly to PI4P than Ex3sk PTB domain (10, 32). Despite similar *in vitro* binding to PI4,5P_2_, the most abundant acidic PI in the plasma membrane, when expressed in cells, only the Ex3in PTB domain exhibits plasma membrane binding (10, 33). While the reason for this has not been determined, the greater affinity of Ex3in PTB for PI4P may be a contributing factor since PI4P plays a major role in establishing negative charges at the plasma membrane and promotes electrostatic binding of polybasic proteins (34, 35). In addition, the exon 3 sequence includes six hydrophobic amino acids which may further stabilize the protein-lipid interaction through the lipid acyl chains.

Localization of *Drosophila* NUMB to cortical membranes is determined by sequences within the N-terminal 76 amino acids that flank the PTB domain including a 30 amino acid cluster of basic and hydrophobic residues (BH) that binds phospholipids and cortical membranes but is not conserved in vertebrate NUMB(see Fig. 2*A*) (36, 37, 38). Thus while NUMB localization to the cortical membrane is conserved between invertebrates and vertebrates, the molecular mechanisms are distinct: whereas the adjacent BH region is required for membrane localization of *Drosophila* NUMB, exon 3 inclusion in vertebrate NUMB regulates membrane localization in an isoform specific manner.

### Exon 9 and the C-terminal region of NUMB: regulation of endocytic protein interactions

The regions outside of the PTB domain in the NUMB protein are predicted to be disordered and include linear tripeptide Asparagine-Proline-Phenylalanine (NPF) and Aspartic acid-Proline-Phenylalanine (DPF) binding motifs (Fig. 1). The two DPF motifs in NUMB are binding sites for α-adaptin and the C-terminal DPF2 motif is conserved in vertebrates, *Drosophila* and *C elegans*. Disruption of the NUMB-AP2 interaction by mutation of the DPF2 motif, or by cellular depletion of AP2, decreases NUMB localization at PM clathrin structures (39). The NPF motif mediates binding to EH-domain family of endocytic proteins, including EPS15, and is conserved in vertebrate and *Drosophila* NUMB.

**Figure 3:**
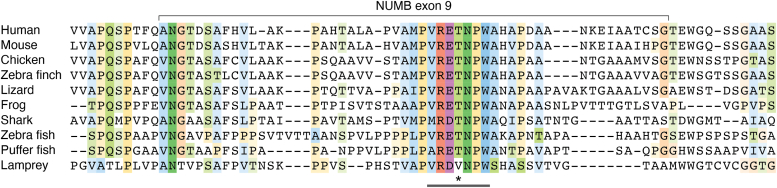
**Species alignment of exon 9 encoded amino acid sequences.** Conserved amino acids are highlighted using the Clustalx color scheme criteria (http://www.jalview.org/help/html/colourSchemes/clustal.html). Within Exon 9 is a highly conserved 16 amino acid region which encompasses a putative basophilic kinase threonine phosphorylation site (indicated by *black* bar). Basal phosphorylation of Numb p72 on the threonine residue within this region has been identified by LC/MS-MS (Krieger, Taylor *et al.* 2015). Amino acid sequences were aligned using Clustal Omega using the default settings using the following sequences: Human (*Homo sapiens*) P49757, Mouse (*Mus musculus*) Q9QZS3, Chicken (*Gallus gallus*) XP_015142420.2, Zebra finch (*Taeniopygia guttata*) XP_030130236.1, Lizard (*Anolis corolinensis*) XP_003214576.1, Frog (*Xenopus tropicalis*) XP_012823284.1, Elephants Shark (*Callorhinchus milii*) A0A4W3K1K3_CALMI, Zebra Fish (*Danio rerio*) NP_001035496.1, Puffer fish (*Takifugu rubripes*) XP_011618128.2, Lamprey (*Petromyzon marinus*) S4R9U0_PETMA. Jalview (178) was used to prepare the figure.

Exon 9 inclusion results in the inclusion of a 48 amino acid sequence within this disordered region, C-terminal to the first DPF motif (Figs. 1, 3). While conserved amongst vertebrates, the exon 9 sequences do not appear to include any known domains or binding motifs. Attempts to isolate proteins which selectively bind this region to date have not identified exon 9 sequence specific interactors (40). Interestingly, protein-protein interactions outside of exon 9 appear to be impacted by the inclusion of the exon 9 encoded sequences. Two endocytic proteins, EPS15 and REPS1, were shown by quantitative mass spectrometry to have greater association with the Ex9in isoform, p72, compared to p66, which lacks Exon 9. Both of these proteins possess an EH-domain which binds the NPF motif in the C-terminus of NUMB. REPS1, unlike EPS15, also bound to the DPF motif (DPF1) upstream of exon 9 insertion, and this interaction is enhanced by inclusion of exon 9 (40).

### NUMB regulation by phosphorylation

Multiple functionally important phosphorylation sites have been identified in NUMB (phosphorylated residues that have been verified experimentally, either by mutational analysis or by mass spectrometry, are shown in Fig. 1). Several studies have demonstrated that phosphorylation regulates NUMB membrane localization during asymmetric cell division (41), the establishment of cell polarity (42), during migration (43, 44) and endocytic recycling (45).

The kinase activity of PKC (39), aPKC (42, 43, 44) and AAK (45) cause the dissociation of NUMB from the plasma membrane and clathrin-coated structures. Mutations of S7, S265 and S284 that prevent phosphorylation, also prevent membrane dissociation in response to kinase activation (42, 41, 43) as well as disrupt asymmetric membrane localization in polarized Madin Darby Canine Kidney (MDCK) cells (42, 46). This is likely, in part, due to stimulated dissociation from AP2 and/or other endocytic proteins since it has been demonstrated that hyper-phosphorylation of NUMB in cells treated with Calyculin A, an inhibitor of protein phosphatases 1 and 2A (40), or phosphorylation of NUMB at S7, S265 and S284 by aPKC (43, 46) and CamK1 (47, 48) decrease its association with AP2. Phosphorylation also inhibits the association of NUMB with endocytic proteins EPS15, REPS1, RalBP1 and AP1B1 (40) as well as plasma membrane receptors β1 and β3 integrin (43) and E-cadherin (46).

Within the exon 9 sequence, the ELM (Eukaryotic Linear Motif) prediction tool identifies a potential basophilic kinase phosphorylation site (VRET^396^NPW; see Fig. 3 showing species alignment of exon 9). Indeed, phosphorylation of T396 within this motif was identified by mass spectrometry (49) suggesting it could provide a means for isoform-specific regulation. Phosphorylation of NUMB (p72) at T79 was also identified by mass spectrometry (49). Although conserved between isoforms, the positioning of T79 adjacent to the 11 amino acid insert (Exon3) and within the residues that mediate an exon 3 dependent protein-protein interaction (Fig. 2) suggests that phosphorylation at this site could sterically influence exon 3-mediated lipid and/or protein interactions (18). While phosphorylation regulates NUMB localization as well as interactions with proteins and lipids it remains unknown whether it does so in an isoform dependent manner.

## NUMB isoforms and endo-lysosomal trafficking

**Figure 4:**
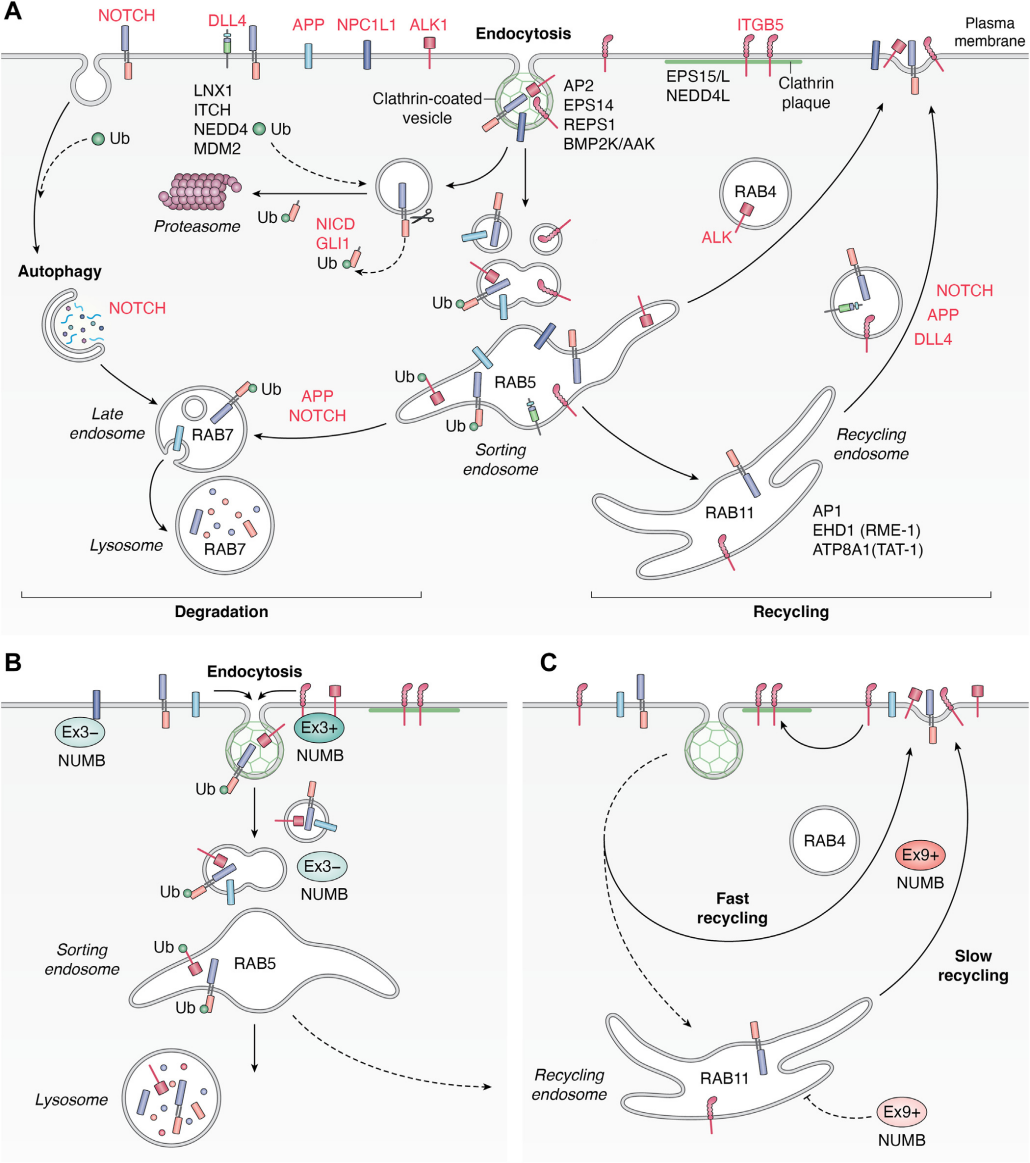
**Numb isoforms and endo-lysosomal trafficking.***A*, schematic representation of the endocytic trafficking pathways and the NUMB-associated proteins/receptors that are discussed in the text. Endocytic trafficking regulates the expression of cell surface receptors that are internalized at the plasma membrane (PM) *via* clathrin-coated vesicles into RAB5 positive endosomal vesicles where they are sorted for degradation in lysosomes, or recycled back to the PM. The balance of these pathways determines the level of expression of receptors at the PM and thus their availability for activation. Evidence suggests that NUMB influences receptor recycling within both a fast route involving RAB4 GTPase positive vesicles, and a slow route that traffics receptors *via* the RAB11 positive recycling endosome. Autophagy, an alternative degradative trafficking pathway utilized by the Notch receptor, is also influenced by loss of NUMB. *B* and *C*, the current evidence which supports unique roles for NUMB isoforms expressing exon 3 (*B*) and exon 9 (*C*). Ex3+ (exon 3 included) NUMB isoforms exhibit greater PM-association than Ex3- (exon 3 skipped) NUMB. This likely promotes its PM function through enhanced association with surface receptors and proteins involved in endocytosis and trafficking, including E3-ligases which mediate protein ubiquitination, marking receptor cargo for sorting into late endosomal vesicles and lysosomes. In some contexts, Ex3-isoforms may also facilitate receptor internalization. Ex3- NUMB contributes to vesicle fusion and transport of cargo to sorting endosomes and promotes trafficking towards degradation. *C*, while exon 9 skipped (Ex9-) isoforms have a conserved role in inhibiting cargo recycling to the PM *via* the slow recycling pathway, possibly through inhibition of other regulatory trafficking proteins, exon 9 included (Ex9+) isoform expression favors recycling of some receptor cargo (eg ALK) to the PM *via* sorting towards the fast recycling route.

Endocytic protein trafficking is a highly regulated process involving the vesicular transport of proteins within the cell (Fig. 4). This pathway determines the localization and expression of transmembrane protein cargo (e.g. receptors and adhesion proteins) at the cell surface by regulating their internalization (endocytosis) into early endosomal vesicles where they are then sorted for either degradation in lysosomes, or recycled back to the plasma membrane (See Fig. 4 for a schematic representation) (reviewed in (50, 51)). NUMB possesses features characteristic of endocytic adaptor proteins: two DPF motifs and an NPF motif which mediate interactions with the endocytic proteins AP2 and EPS15 respectively, and a PTB domain which can bind cargo proteins. These features are conserved between the invertebrate form and the four isoforms of vertebrate NUMB. While involvement in endo-lysosomal trafficking is a conserved function of NUMB, there is evidence to suggest unique functions of the NUMB isoforms within this pathway. In the following section we outline what is known about the role of invertebrate NUMB, which exists as a single form, and then review the studies in higher organisms which ascribe functions to the individual isoforms expressed in vertebrates.

### Function of invertebrate NUMB in endocytic trafficking

Much of what is known about the trafficking functions of NUMB in invertebrates derives from studies addressing the regulation of Notch activity in asymmetrically dividing stem cells during *Drosophila* development. Unequal partitioning of dNUMB into daughter cells inhibits Notch function in the receiving cell by preventing its accumulation at the cortical cell-cell junctions where it is normally activated by the Notch ligand, Delta (for reviews see (52, 53)). In general, the expression of receptors such as Notch at the cell surface, where they are functionally active, is determined by both protein synthesis and endo-lysosomal trafficking. The latter process regulates their loss from the membrane (*i.e.* internalization by endocytosis), the return of internalized protein to the membrane (*i.e.* recycling), and their degradation within lysosomes. A number of studies have shown that during asymmetric cell division the presence of dNUMB in the receiving daughter cell suppresses endocytic recycling of Notch and its coreceptor, Sanpodo, resulting in their degradation in lysosomes (54, 55, 56). Experiments following the localization of fluorescence-tagged Sanpodo and Notch proteins demonstrated that dNUMB inhibits the recycling of Notch/Sanpodo *via* the clathrin adaptor protein AP-1 (55, 57, 58). AP-1 is involved in positively regulating recycling of Notch/Spdo from the recycling endosomes to the basolateral membrane (58, 59). The asymmetric accumulation of NUMB therefore is thought to inhibit AP-1, preventing the recycling and subsequent activation of Notch/Sanpodo. In support of this, Cotton *et al.* (2013) demonstrated that dNUMB can immunoprecipitate co-expressed AP-1 from S2 cells. However, how dNUMB interaction with AP-1 inhibits its activity is currently unknown.

The *C. elegans* ortholog of NUMB, Num-1, inhibits vesicle trafficking and causes the formation of an enlarged endosomal compartment, suggesting that Num-1 may also block vesicle recycling to the plasma membrane. It is unclear how this occurs but may involve inhibition of Rme-1, the orthologue of mammalian recycling associated protein EHD1 (60), or/and the phospholipid transporting ATPase tat-1 (61), mutants of which cause a block in early endosomal transport, possibly by preventing phospholipid-dependent tubulation (62).

In summary, despite the presence of binding motifs for proteins involved in early clathrin-mediated endocytosis, the single invertebrate NUMB isoform does not appear to influence the formation of early endocytic vesicles nor the rate of membrane cargo internalization (54, 55, 63). Rather, in invertebrate model systems, NUMB functions to disrupt vesicle recycling and promote the degradation of membrane receptors such as Notch.

### Conserved functions of vertebrate NUMB in endocytic trafficking

How the evolution of NUMB isoforms has impacted its role in vesicular trafficking is only beginning to be understood. To date, studies examining the effect of loss of all isoforms, together with experiments over-expressing individual isoforms, generally support findings in *Drosophila* and *C elegans* in which NUMB regulates the surface expression of proteins by influencing intracellular sorting to recycling or degradative pathways. For instance, experimental depletion of total NUMB has been consistently shown to alter the intracellular accumulation of membrane cargo (43, 64, 65, 66, 67, 68), often in the absence of changes in endocytosis (64, 68). Several of these studies provide evidence that the differences in cargo accumulation (*e.g.* Notch, Dll4) caused by NUMB loss is the result of enhanced recycling to the plasma membrane indicating that vertebrate NUMB also acts to inhibit recycling (64, 68).

Whether vertebrate NUMB influences sorting decisions through similar mechanisms involving AP-1, as described for *Drosophila* NUMB (55, 57, 58), or by influencing vesicle fission through a tat-1 orthologue, as in *C. elegans* (61), remains to be determined. AP-1 is a heterotetrameric complex (γ,β1,μ1,σ1) which in mammals exists in two functionally distinct forms: AP-1A (μ1A) that localizes to the *trans*-Golgi network (TGN) and early endosomes, and the epithelial specific AP-1B (μ1B) that also localizes to the recycling endosome (69, 70). Endogenous NUMB has been co-localized with the gamma subunit of AP-1 at the TGN (71), and there is evidence of a NUMB complex with both the gamma and beta subunits (55, 68). In one study that addressed isoform specificity, AP-1 binding appears to be independent of exon 9 since a quantitative comparison of AP-1 beta subunit binding to NUMB p66 (Ex9sk) and p72 (Ex9in) isoforms revealed no difference (40).

The negative regulatory effect of *C. elegans* num-1 on tat-1 (described above) may also be conserved in vertebrates through an association of NUMB with the mammalian orthologues of Rme-1 and tat-1: EHD1 and ATP8A1 respectively. ATP8A1 is located at the recycling endosome where it translocates phosphatidyserine (PS) from the luminal membrane leaflet to the cytosolic leaflet. In mammalian cells, the NUMB-interacting protein EHD1 also localizes to recycling endosomes with GFP-ATP8A1 and this localization is disrupted when ATP8A1 is depleted (72). Depletion of either ATP8A1 or EHD1 causes the accumulation of aberrant recycling endosomes and suppression of cargo recycling to the plasma membrane. NUMB has been demonstrated to bind both EHD1 and the related protein EHD4 and to colocalize with EHD4 at recycling endosomes in HeLa cells (73). Interestingly, the related P4-ATPase, ATP11A, which also translocates PS across membranes (74), appears to preferentially immunoprecipitate NUMB Ex9in from the pancreatic cancer cell lines PANC-1 and SW1990 (75) eluding to differential roles for NUMB isoforms. Further studies are required to confirm if NUMB can bind to and inhibit the function of ATP8A1 (or other related P4-ATPases), and whether NUMB isoforms have distinct activities in this regard.

### Exon 3 inclusion affects cargo sorting within the early endocytic pathways

Although multiple studies have examined the role of a single NUMB isoform on membrane receptor trafficking, few have specifically compared the isoforms. However, evidence to date points to the involvement of exon 3 splicing in cargo sorting early in the endocytic trafficking cycle, either at the point of internalization (endocytosis), or downstream *via* endocytic vesicle formation and targeting for degradation.

It is clear that exon 3 inclusion promotes the association of NUMB with plasma membrane endocytic structures (detailed above), thus ideally positioning it for a role in membrane protein endocytosis. Also, the NUMB PTB domain binds many membrane proteins, including growth factor receptors and integrins (Table 1). This, together with its conserved endocytic protein binding motifs (NPF and 2 DPF), provides a putative mechanism for bringing together membrane cargo with the endocytic proteins required for their endocytosis (76). While several studies have concluded that neither overexpression of NUMB p65 (71) or p66 (64), nor knockdown of NUMB (68) alters the rate of internalization of EGFR, Notch, or DLL4, respectively, others suggest a receptor- or/and context-dependent role in endocytosis. For example, in MCF7 epithelial cells cultured in conditions where E-cadherin is free in the membrane, endocytosis is inhibited by NUMB depletion (46). In contrast, in polarized MDCK epithelial cells, where E-cadherin is tethered by homotypic interactions at cell-cell adhesions, E-cadherin endocytosis is enhanced when NUMB is depleted (66). The opposite effects of NUMB depletion observed in these studies possibly reflect independent functions of NUMB in distinct E-cadherin internalization pathways.

**Table 1:** Proteins and phospholipids which bind NUMB PTB domain

Interacting protein	NUMB protein							Reference
	Ex3out PTB	Ex3in PTB	p65	p66	p71	p72	Other∧	
Membrane								
ALK	Yes	Yes	-	-	-	p72	-	(92)
APP	-	-	p65	p66	p71	p72	-	(179)
			-	p66	-	-	-	(97)
			p65	p66	p71	p72	-	(78)
E-Cadherin	-	-	p65	-	-	-	-	(180)
	weak	-	P65	-	-	-	-	(46)
EAAT3	Yes	-	-	-	-	-	-	(181)
EPHB2	Yes	-	p65	-	-	-	-	(182)
ERBB2	Yes	-	-	-	-	-	-	(183)
	-	-	P65	P66	P71	P72	-	(98)
FGR	Yes	-	-	-	-	-	-	(183)
FLT3	Yes	-	-	-	-	-	-	(183)
ITGB1	-	weak	-	-	-	-	-	(184)
	Yes	-	p65	-	-	-	-	(43)
	-	No	-	-	-	-	-	(185)
ITGB3	-	Yes	-	-	-	-	-	(184)
	Yes	-	p65	-	-	-	-	(43)
	-	No	-	-	-	-	-	(185)
ITGB5	-	Yes	-	-	-	-	-	(184)
	-	Yes	-	-	-	-	-	(185)
*d*-NIP	-	-		-	-	-	*d*-numb	(186)
NPC1L1	Yes	No	-	-	-	-	-	(67)
	Yes	-	p65	-	-	-		(77)
OPO/OFCC1	-	Yes	-	-	-	p72	-	(187)
RET	Yes	-	-	-	-	-	-	(183)
ROS1	Yes	-	-	-	-	-	-	(183)
Sanpodo	-	-	-	-	-	-	*d*-numb	(55, 188)
	-	-	-	-	-	-	*d*-numb PTB	(189, 190, 191)
p-Selectin	Yes	-	-	-	p71	-	-	(192)
*Ce*-TAT-1	-	-	-	-	-	-	*Ce*-Num1 PTB	(61)
TRKB	Yes	-	p65	-	-	-	-	(44)
VEGFR2	Yes	-	-	-	-	-	-	(193)
VEGFR3	Yes	-	-	-	-	-	-	(193)
Cytosolic								
AAK	-	Yes	-	-	-	-	-	(45)
BMP2K	-	-	-	p66	-	p72	-	(40)
*d*-NAK	Yes	-	-	-	-	-	*d*-numb PTB	(19, 194)
	yes	-	p65	-	-	-	*d*-numb PTB	(195)
CCM1 (KRIT1)	Yes	Yes	-	-	-	-	-	(32)
CK2a, CK2b	-	-	p65	p66	No	p72	-	(196)
Cortactin	-	-	p65	p66	No	p72	-	(196)
CRMP2/Dpysl2	Yes	-	p65	-	-	-	-	(197)
*d*-CRMP	-	-	-	-	-	-	*d*-numb PTB	(198)
*d*-DRONC	-	-	-	-	-	-	*d*-numb PTB	(177)
EFA6	-	Yes	-	-	-	p72	-	(199)
ITCH	-	-	-	p66	-	-	-	(96)
	-	-	-	P66	-	-	-	(101, 102)
LNX1	No	Yes	No	p66	No	p72	-	(20, 23)
	Yes	Yes	-	-	-	-		(22)
LNX2	Yes	-	p65	p66	-	-	-	(195, 200)
MDM2	No	Yes	-	-	No	p72	-	(18)
	-	-	p65	-	-	-		(201)
	-	-	p65	p66	p71	p72	-	(24)
	-	Yes	-	-	-	p72	-	(202)
NBD (KANK family)	Yes	Yes	-	-	p71	p72	-	(203)
NEDD4-1	Yes	-	p65	-	-	-	-	(100)
*d*-Notch ICD	-	-	-	-	-	-	*d*-numb PTB	(204)
Notch1 ICD	Yes	-	p65	-	-	-	-	(205)
Notch1 ICD	-	-	P65	P66	P71	P72	-	(99)
mPAR3	Yes	-	p65	-	-	-	-	(43)
PICALM	-	Yes	-	-	-	p72	-	(206)
Ce-PKC3	-	-	-	-	-	-	Ce-Num-1 PTB	(7, 207)
*d*-PON	No	-	-	-	-	-	*d*-numb PTB	(19)
SIAH	-	-	p65	-	-	-	-	(208)
SKP1	-	Yes	-	-	-	p72	-	(209)
TP53	Yes	Yes	p65	-	-	-	-	(210)
	-	-	-	-	-	-	endog	(211)
	-	-	p65	-	-	-	-	(212)
	-	-	-	-	-	p72	-	(41)
Phospholipid								
PI(3,4)P2	Yes	-	-	-	-	-	-	(32)
PI(3,5)P2	Yes	-	-	-	-	-	-	(32)
PI(4,5) P2	Yes	Yes	-	-	-	-	-	(10)
PI(3,4,5)P3	Yes	Yes	-	-	-	-	-	(10, 32)
PI(3)P	Yes	Yes	-	-	-	-	-	(10)
PI(4)P	No	Yes	-	-	-	-	-	(10)

(−): not tested; (Yes): interaction; (No): no interaction; (ˆ) non-mammalian forms of NUMB: *Drosophila* (d), *C. elegans* (ce).

NUMB depletion also prevents cholesterol-stimulated endocytosis of the cholesterol transport protein, NPC1L1 (67, 77). Experiments comparing overexpression of each isoform in NUMB-depleted cells showed that only the Ex3sk NUMB isoforms (p71 and p65) can rescue NPC1L1 endocytosis. Consistent with this, only Ex3sk isoforms bind NPC1L1 (67). NUMB p65 facilitates NPC1L1 endocytosis through its interaction with AP2/clathrin (77) and NUMB mutants defective in binding AP2 do not rescue (67). This study suggests that Ex3sk isoforms preferentially link NPC1L1 to endocytic machinery to facilitate the stimulated endocytosis of NPC1L1.

The presence or absence of exon 3 also determines the intracellular accumulation and sorting of Amyloid Precursor Protein (APP). However, in contrast to NPC1L1, APP can bind all NUMB isoforms similarly, and exon 3 skipped isoforms appear to influence the endocytic sorting of cargo towards recycling or degradation. Over-expression of Ex3sk NUMB isoforms in PC12 cells promoted the accumulation of APP in enlarged RAB5-positive early endosomes and this correlated with an increase in both total cellular APP (unrelated to protein synthesis) and APP expressed at the cell surface (78). Ex3in isoforms (p66, p72) had the opposite effect (78). Kyriazis *et al.* 2008 (78) postulated that Ex3sk NUMB isoforms regulate the post-endocytic sorting of APP towards a RAB5 positive endosomal compartment and recycling to the PM. This pathway is discussed further below.

Another study looking more specifically at the formation of RAB5-positive endosomal vesicles, downstream of endocytosis, concluded that NUMB isoforms that lack exon 3 may regulate the formation of these structures (79). Shao *et al.* (2016) (79) observed by immunofluorescence staining with a marker of early endosomes, EEA1, that knockdown of NUMB in MCF7A cells caused an increase in the number of early endosomal vesicles. This effect could be rescued by overexpression of the Ex3sk isoforms of NUMB (p71 and p65), but not Ex3in isoforms (p72 and p66). Using time-lapse imaging to visualize vesicle docking and fusion of early endosomal vesicles, either loaded with fluorescently-tagged cell surface receptor ligands LDL or transferrin, or labeled with RAB5-RFP, a marker for early endosomes, they observed that knockdown of NUMB caused a decrease in the number of docking/fusing endosomes. These observations suggest that loss of NUMB inhibits the vesicle fusion step, upstream of sorting towards degradation or recycling. Furthermore, Shao *et al.* 2016 (79), provide evidence that this process may be linked to recruitment of MON1b, part of the MON1-CCZ1 RAB7 GEF complex involved in the maturation of late endosomal vesicles to lysosomes. It is not clear how NUMB interacts with early endosomes, though evidence suggests that this function depends on the exon 3-excluded form of the PTB domain (79). Together, these studies indicate that NUMB regulates the internalization of membrane cargo and sorting of early endosome cargo to protein recycling and lysosomal pathways in an exon 3-dependent manner.

### Exon 9 modulates NUMB association with the endocytic machinery to regulate membrane receptor surface expression

NUMB proteins bind the endocytic adaptor proteins EPS15 and its functional paralogue EPS15L1 (EPS15R). These closely related scaffold proteins possess 2 ubiquitin interaction motifs (UIM) which bind ubiquitinated proteins, multiple AP2-interacting DPF motifs, and 3 EH domains which recognize the NUMB NPF motif (71). EPS15/EPS15L1 are recruited early in the formation of clathrin-coated vesicles (80, 81) and have been demonstrated to act both redundantly and non-redundantly in endocytosis (82, 83). Several studies have implicated NUMB-EPS15- and -EPS15L1 interactions in the regulation of clathrin-mediated internalization of cargo (84, 85). For example, NUMB p72 binds both EPS15/EPS15L1 and the membrane receptor EPHB2, forming a complex which is enhanced by stimulation with soluble ephrinB1 ligand and appears to mediate EPHB2 internalization *via* clathrin coated pits (85) Similarly, in primary rat dorsal root ganglion neurons, a NUMB-EPS15-NEDD4L complex is recruited by the NUMB PTB- binding protein, CRMP2, to the voltage-gated sodium channel NaV1.7, facilitating its internalization and thus decreasing sodium flux across the plasma membrane (84). Although EPS15 and EPS15L1 are predicted to bind all isoforms of NUMB *via* the conserved C-terminal NPF motif, quantification of protein complexes associated with NUMB p66 and p72 using selected reaction monitoring (SRM) mass spectrometry showed that EPS15 exhibits preferential association with isoforms that include exon 9 (40). This was confirmed in intact cells using a proximity ligation assay (PLA) to compare colocalization of NUMB p66 and p72 with EPS15 at the PM of HEK293T cells. Thus the differential association of NUMB isoforms with endocytic proteins may serve to nucleate complexes that facilitates entry of cargo into the endocytic pathway.

Integrins are transmembrane, heterodimeric receptors that bind extracellular matrix (ECM) proteins, linking these to intracellular signaling pathways involved in many cellular functions, including migration, cell growth and survival, and development (86). NUMB binds multiple integrin family members *via* its PTB domain (Table 1), including β5 integrin (ITGB5), a component of large clathrin-containing, adhesive structures at the plasma membrane (variously referred to in the literature as clathrin plaques, reticular adhesions, flat clathrin lattices) (reviewed in (87)). NUMB has localized to clathrin plaques together with NUMB-associated endocytic proteins, EPS15, EPS15L1 and the E3 ubiquitin-ligase NEDD4L (88, 89, 90). In PA-JEB/B4 keratinocytes, wild type ITGB5 clusters in flat clathrin lattices at the ventral surface of the cell, a process that is dependent on Ca^2+^ and the ECM ligand vitronectin. Using a proximity-biotinylation assay, BirA∗ biotin ligase tagged-ITGB5 was demonstrated to label endogenous NUMB in these cells and this was dependent on the presence of the ITGB5 PTB-binding motif. Knock down of either NUMB or EPS15/EPS15L1 resulted in decreased ITGB5 clustering suggesting that NUMB together with EPS15L1 regulates ITGB5 clustering within flat clathrin lattices (90). Furthermore, a study examining the effect of NUMB exon 9-specific deletion on cellular protein networks found decreased surface expression of multiple membrane proteins, including ITGB5 which also exhibited decreased clustering (91). Though not yet validated experimentally, these data, together with the enhanced interactions of NUMB p72 with EPS15 described above, are consistent with a preferential role of NUMB E x 9 isoforms in ITGB5 clustering through interaction with EPS15L1.

Finally, several studies suggest that the NUMB exon 9 included isoforms may also promote rather than inhibit the recycling of cargo to the plasma membrane (91, 92). Wei *et al.* (2019) compared the effects of NUMB isoforms p66 (Ex9sk) and p72 (Ex9in) over expression on the endosomal trafficking of the anaplastic lymphoma kinase (ALK) receptor tyrosine kinase in stably-transfected HEK293T cells (92). Surface biotinylation assays examining the fate of internalized biotin-labelled ALK suggested that p72 and p66 have different effects on ALK trafficking: whereas p66 promotes degradation, p72 promotes recycling of ALK to the membrane. In addition, NUMB p72, but not p66, enhanced accumulation of ALK within fast-recycling vesicles, identified by localization with GFP-RAB4 (see Fig. 4). Thus, the exon 9 included isoform of NUMB (p72) preferentially promotes accumulation of ALK at the plasma membrane through increased transport through the fast recycling pathway. These observations are consistent with a study examining the functional effects of CRISPR-mediated deletion of exon 9 from MDA-MB-468 cells (91). A comparison of the total proteome of control cells, which express all four NUMB isoforms with exon 9 deleted cells, revealed increased abundance of cell surface proteins including growth factor receptors, nutrient transporters and integrins as well as proteins involved in membrane protein recycling (e.g., RAB4A, RAB34 and AP1M1) in cells expressing Ex9in isoforms (91). Together these studies suggest that the exon 9 included isoforms promote recycling, potentially by preventing the inhibitory function of the exon 9 skipped isoforms or through an independent mechanism.

### Role of NUMB isoforms in ubiquitin-dependent degradation pathways

Cellular proteins are degraded by multiple mechanisms, including the endo-lysosome and ubiquitin-proteasome pathways (for reviews of these pathways, see (93, 94, 95)). Covalent attachment of ubiquitin is a common way by which membrane proteins are tagged for sorting towards these pathways. Within the endo-lysosomal pathway, loss of NUMB expression is associated with decreased degradation of Notch1 (64) while expression of the Ex9sk isoform (p66) promotes Notch1 ubiquitination and lysosomal degradation (64, 96). Similarly, loss of NUMB expression decreased accumulation of membrane cargo such as APP (78, 97) and ERBB2 (98) in LAMP1-positive vesicles of the lysosomal degradative pathway. Over-expression of NUMB p66 also decreases the accumulation of total APP in PC12 cells, consistent with enhanced degradation while overexpression of NUMB Ex3sk p65, had the opposite effect, consistent with a role for exon 3 inclusion in sorting cargo towards lysosomal degradation (78). In support of this, pre-treatment with inhibitors of lysosomal function restored APP accumulation in p66-expressing cells, but had no effect on APP levels in p65-expressing cells (78).

As described above, Wei *et al.*, used surface protein biotinylation assays to quantify ALK at that PM, and showed that NUMB p66, but not p72 overexpression caused a decrease in surface expression of ALK that could be prevented by inhibiting lysosomal degradation (92). Thus, the presence of exon 3 may be required for recognition of cargo destined for lysosomal degradation, while exon 9 inclusion may override exon 3 preventing degradation by promoting recycling to the plasma membrane (92). These results reenforce the importance of side-by-side comparisons of the NUMB isoforms to inform function.

In addition to PM cargo, NUMB also mediates the degradation of cytoplasmic proteins *via* the ubiquitin-proteosome pathway. Such proteins include the tumor suppressor p53, GLI1 transcription factors, and the Notch intracellular domain (NICD). NUMB promotes E3-ligase mediated ubiquitination of NICD, which targets it for degradation by the proteasome (96, 99). A single isoform study showed that NUMB (p66) overexpression enhances ubiquitination of NICD, and decreased the accumulation of endogenous NICD when Notch was activated (96).

The association of NUMB with ubiquitin E3 ligases such as ITCH, NEDD4 (100), MDM2 and LNX1 is important for its functions within lysosomal and proteasomal degradation. For example, NUMB p66 PTB (Ex3in) directly binds the E3 ligase ITCH (96, 101, 102) and when co-expressed they act synergistically to increase Notch ubiquitination (96). Similarly, NUMB (p66) binding activates ITCH E3 ligase activity towards the Hedgehog transcription factor, GLI1 facilitating its ubiquitination and degradation (101, 102).

Differential association of NUMB isoforms with E3 ligases is a potential mechanism for regulating ubiquitin-mediated degradation. Indeed, NUMB PTB domain binding to two E3 ligases, MDM2 and LNX1, is enhanced by the inclusion of exon3. Early studies showing that NUMB regulates MDM2-mediated degradation of p53 reported that all isoforms of NUMB co-immunoprecipitated with MDM2 (24). However, using more sensitive fluorescence polarization assays, it was later shown that Ex3in PTB domain exhibited higher affinity binding to MDM2 than did Ex3sk PTB (18). The interaction of NUMB with another E3 ligase, LNX1 (20), is also enhanced by the inclusion of exon 3, which results in the isoform specific degradation of NUMB Ex3in itself. Whether the interaction between NUMB and ITCH is similarly regulated by exon 3 splicing is unknown, but would be consistent with Ex3in isoform specific regulation of cargo ubiquitination and sorting to the degradative pathway.

In summary, NUMB proteins play a complex role in the surface expression of many receptors and adhesion proteins by regulating trafficking through endo-lysosomal transport pathways. While this core function is evolutionarily conserved, the differential binding of vertebrate NUMB isoforms to cargo proteins, membrane phospholipids and/or the endocytic machinery as well as isoform-specific effects on endocytic vesicle maturation serve to fine-tune this function. The evidence to date suggests that exon 3 may influence the function of NUMB early in endocytosis and cargo sorting towards degradation, whereas exon 9 promotes the recycling of cargo and possibly its stabilization at the membrane. Since most cell types examined express multiple NUMB isoforms the fate of membrane cargo is likely to be determined by the balance of isoform expression.

## Regulation of NUMB isoform expression in development and disease

Homozygous deletion of NUMB is embryonic lethal in mice at day 11.5, with defects in neurogenesis, angiogenic remodeling, and placenta formation (103, 104). While deletion of NUMBL has no observable effects on mouse development, combined deletion of NUMB and NUMBL results in embryonic lethality at day 9, earlier than NUMB alone, suggesting that NUMB and NUMBL may have some overlapping functions, particularly during neurogenesis (105, 106). Conditional deletion of NUMB revealed essential roles in neurogenesis (107, 108), heart development (98, 109), mammary gland development (110, 111), and retinal development (112). While these studies clearly define the essential role of NUMB during development, the importance of individual isoforms is less well understood. However, the temporally regulated and tissue-specific expression patterns described below is supportive of distinct developmental functions for the NUMB protein isoforms and evidence that this is disrupted in disease states.

### Developmental and tissue-specific expression of NUMB isoforms

The expression pattern of NUMB splice variants and protein isoforms differs across tissues and cultured cell lines. An early study using NUMB isoform-specific antibodies confirmed distinct cell and tissue type expression of the four major protein isoforms derived from exon 3 and exon 9 skipping and inclusion (10). The differences in protein isoform expression between tissues observed were consistent with cell type-specific and developmental regulation of NUMB splicing. Indeed, this is supported by experimental evidence using mouse embryonal carcinoma P19 cells as a neural differentiation model: retinoic acid-induced differentiation of P19 cells results in the switch in NUMB isoform expression from predominantly Ex9in to exclusively Ex9sk isoform expression in differentiated cells (10, 113). This pattern of isoform switching during mouse development has been observed in neural tissues (114, 115, 116, 117), rodent pituitary gland (118), rat auditory epithelium (119), pancreas (120) and erythroid CD34+ hematopoietic stem cells (121) (Table 2). Studies examining the effect of ectopic or over expression of individual isoforms in *Drosophila* (116, 122) and mouse neural stem cells (114, 116, 123) support distinct functions of Ex9in and Ex9sk NUMB isoforms in development and differentiation.

**Table 2:** Developmental changes in NUMB exon 9 and exon three

Tissue or cell type	Exon inclusion		Reference

Human brain development	↓E9	-	(116)
Rat brain development			
Mouse cerebral cortex development	↓E9	↓E3	(114)
Retinoic acid (RA) induced differentiation of mouse embryonic carcinoma p19 cells	↓E9	↓E3	(10)
	-	↓E3	(114)
	-	↓E3	(123)
Mouse pancreas lineage specification			(120)
Endocrine:	↓E9		
Exocrine:	↑E9		
Zebrafish embryo from 64 cells to 5 days		↑↓	(33)
Mouse retina development	↓E9		(115)
From adult mouse testis (predominantly p71), to PN8 testis, to adult germ cell-depleted testis	↓E9	↑↓	(135)
Rat cochlear development	↓E9	↓E3	(119)
Mouse pituitary development	↓E9	↓E3	(118)
Chicken embryo development	↓E9		(117)
Hematopoiesis			
hESC(H1) differentiation to CD31 + CD34+ EPC	↓E9	-	(127)
Erythroid Development			
Human CD34+ hematopoietic stem cells	↓E9	-E3	(121)
Murine erythroleukemia cells (MELC)	↓E9	-E3	

In normal human tissues, analysis of NUMB exon 9 expression levels using semiquantitative RT-PCR, found predominantly exon 9 skipped mRNA expression in most tissues, while a subset of tissues had a higher ratio of exon 9 included mRNAs (breast, colon, kidney, pancreas, rectum, and testis) (124). Analysis of publicly available RNA sequencing data comparing NUMB mRNA transcripts across multiple tissue types found that in human embryonic stem cells (ESC) and induced pluripotent stem cells (iPS), the exon-inclusion ratio (percentage splice in index, or PSI) for exon 9 is greater than 50% and relative exon 9 inclusion was higher in a subset of tissues including testis, pancreas and gastrointestinal tract (125). In contrast, in adult tissues such as brain, pituitary, and hematopoietic cells, exon 9 inclusion is less than 25%, and heart, adipose, breast, lung, adrenal, and gynecologic tissues exhibit negligible exon 9 inclusion. These observations are in agreement with murine studies described above that showed a higher ratio of exon 9 inclusion in progenitor and stem cell populations. NUMB Ex3in transcripts also exhibited a tissue-specific pattern of expression, with notably high exon 3 inclusion in ESC and iPS suggesting that NUMB p72 is the most abundant isoform in these cells while in adult brain and pituitary gland, as well as immune and hematopoietic cells, exon 3 inclusion is lower than 25% (125).

### NUMB isoform expression during development is determined by multiple RNA splicing regulators

Tissue-specific alternative splicing is determined, in part, by developmentally regulated splicing factors and other RNA-binding proteins (RBP) (126). A number of these have been demonstrated to alter NUMB splicing, particularly exon 9 splicing, including Rbfox3 (117), SRSF family members (113, 127, 128), RBM4/5/10 (123, 129), PTBP1 (113, 130) and QKI-5 (131, 132) (Table 3 shows splicing regulators and their effect on NUMB alternative splicing). For example, in the P19 cell neuronal differentiation model, the knockdown of RBM4 prevents the splicing switch from dominant exon 9 inclusion to skipping in differentiated cells (123). In the chick embryo, NUMB undergoes isoform switching regulated by the RNA-binding Fox (Rbfox) protein family member, RbFox3 (117). Knockdown of RbFox3, in chick neural tube reduced exon 9 skipping while forced expression enhanced exon 9 inclusion. Kim *et al.* (2013) confirmed that RbFox3 binds UGCAUG elements within the intronic sequence immediately upstream of exon 9 in NUMB pre-mRNA (117).

**Table 3:** Regulators of NUMB alternative splicing

Splicing regulator	Type	Exon inclusion	Reference
Rbfox3	Splicing Factor	↓E9	(117)
Rbfox2	Splicing Factor	↓E9	(139)
PTBP1	Splicing Factor	↑E9	(113, 130)
SRSF1	Splicing Factor	↓E9	(113)
SRSF2	Splicing Factor	↑E9	(127)
SRSF3	Splicing Factor	↑E9	(128)
TDP43	Splicing Factor	↑E9	(128)
RBM4	Splicing Factor	↓E9 ↑E3	(123)
RBM5	Splicing Factor	↑E9	(129)
RBM6	Splicing Factor	↑E9	(129)
RBM10	Splicing Factor	↓E9	(129, 157, 158)
QKI-5	Splicing Factor	↓E9	(131, 132, 134)
NSrp70	Splicing Factor	↓E9	(213)
ESRP1	Splicing Factor	↑E9	(214)
PQBP1	Splicing Factor	↑E9	(215)
ELK1	Transcription factor	↑E9	(130)
MYC	Transcription factor	↑E9	(130)
ERK/MAPK	Kinase	↑E9	(113)
SRPK2	Kinase	↑E9	(139)
miR-355	MicroRNA	↑E9	(158)

[↑E9, ↑E3] increased exon inclusion; [↓E9] decreased exon 9 inclusion.

Multiple members of the family of serine/arginine (SR)-rich splicing factors regulate NUMB exon 9 splicing, including SRSF1, SRSF2 as well as SRSF3 (113, 127, 128). In a NUMB exon 9 minigene splicing reporter assay, whereas SRSF1 expression promotes exon 9 skipping (113), SRSF2 increases exon 9 inclusion (127). In agreement, Li *et al.* demonstrated that in human embryonic stem cells and mesodermal cells, SRSF2 promotes inclusion of NUMB exon 9. During differentiation to endothelial progenitors. SRSF2 is downregulated, Ex9in transcripts are replaced by NUMB Ex9sk transcripts (127). Using the RBPmap prediction algorithm, these authors identified putative SRSF2 binding motifs required for NUMB exon 9 splicing in response to SRSF2 expression (127). Similarly, a switch from exon 9 inclusion to exon 9 skipping occurs during the induced differentiation of human CD34+ hematopoietic stem cells and murine erythroleukemia cells (121), coinciding with increased expression of SRSF1 (133).

Finally, the RNA-binding protein Quaking (QKI) plays an essential role during embryonic and postnatal development. QKI-5 protein increases during muscle stem cell differentiation and regulates alternative splicing events in a number of cell polarity proteins and markers of differentiation, including NUMB (131, 132, 134). QKI-5 promotes NUMB exon 9 skipping through its ability to recognize and bind to two binding sites flanking the 3′ splice site of intron 9 and competing with another splicing factor, SF1, which also binds to one of the two splice sites.

Splicing of exon 3 also shows a pattern of regulation during development though much less is known about the factors involved. For example, in the mouse P19 cell differentiation model, exon 3 inclusion declines in response to retinoic acid induced neuronal differentiation (10, 123). Similarly, reduced exon 3 inclusion has been observed during development of mouse testis (135), mouse cerebral cortex (114), rat auditory epithelium (119) and mouse pituitary (118) (Table 2). Despite evidence for exon 3 splice switching in several tissues, only RBM4 has be demonstrated to modulate exon 3 splicing. Co-expression of RBM4 and an exon 3 minigene reporter construct resulted in increased exon 3 inclusion in P19 and HEK293T cells (123).

### Deregulation of NUMB exon 9 alternative splicing in cancer

Mis-regulation of NUMB alternative splicing is broadly associated with many cancer types and impacts several cellular processes associated with tumor progression (Table 4) (136). Indeed, two independent studies, using unbiased exon arrays to identify alternative splicing events in non-small cell lung carcinoma (NSCLC) *versus* normal adjacent tissue, identified NUMB exon 9 inclusion as one of the most frequent tumor-associated splicing events (124, 137). In contrast, cancer associated changes in splicing of exon 3 were not observed in these studies.

**Table 4:** Changes in *NUMB* exon 3 and exon 9 splicing in cancer

Cancer type	Sample size	Exon inclusion		Reference
Cervix	CSCC	-	↑E9	(138)
	CESC	-E3	↑E9	(91)
Lung	NSCLC: LUAD, SCC	-	↑E9	(137)
	NSCLC: LUAD	-E3	↑E9	(124)
	LUAD, LUSC	-E3	↑E9	(91)
Colon	Colon	-	↑E9	(124)
	COAD	-	↑E9	(130)
	CRC	-	↑E9	(216)
Breast		-	↑E9	(124)
Breast cancer subtypes	Luminal A	↓E3	↑E9	(91)
	Luminal B	↓E3	↑E9	(91)
	Basal-like, Her2-enriched, Normal-like	-E3	↑E9	(91)
	BRCA	↓E3	↑E9	(91)
Liver	HCC	-	↑E9	(139)
	HCC	-	↑E9	(139)
	HCC	-	↑E9	(217)
	LIHC	-E3	↑E9	(91)
Bladder	urothelial bladder tumor	-	↑E9	(141)
	BLCA	-E3	↑E9	(91)
Pancreas	pancreatic ductal tumor	-	↑E9	(218)
	PAAD	-E3	↑E9	(91)
	PAAD	-	↑E9	(75)
Medullo-blastoma subtypes	Wnt, Shh, G3, G4 Medulloblastoma	-	↑E9	(142)
Uterine	UCEC	-E3	↑E9	(91)
Prostate	PRAD	-E3	↑E9	(91)
Stomach	STAD	-E3	↑E9	(91)
Thyroid	THCA	↓E3	-E9	(91)
Kidney	KICH	↓E3	-E9	(91)
	KIRP	-E3	-E9	(91)
	KIRC	-E3	↓E9	(91)

[-E3, -E9] no change; [↑E9] increased exon 9 inclusion; [↓E3, ↓E9] decreased exon inclusion;[-] not tested.

BLCA, bladder urothelial carcinoma; BRCA breast invasive carcinoma; CESC, cervical squamous cell carcinoma and endocervical adenocarcinoma; CHOL, cholangiocarcinoma; COAD, colon adenocarcinoma; ESCA, esophageal carcinoma; KICH, kidney chromophobe renal cell carcinoma; KIRC, kidney renal clear cell carcinoma; KIRP, kidney renal papillary cell carcinoma; LIHC, liver hepatocellular carcinoma; LUAD, lung adenocarcinoma; LUSC, lung squamous cell carcinoma; PAAD, pancreatic adenocarcinoma; PCPG, pheochromocytoma and paraganglioma; PRAD, prostate adenocarcinoma; READ, rectum adenocarcinoma; STAD stomach adenocarcinoma; THCA thyroid carcinoma; UCEC, uterine corpus endometrial carcinoma.

Increased exon 9 inclusion has been reported in multiple primary tumor types including cervical squamous cell carcinoma (CSCC) (138), non-small cell lung cancer (NSCLC) (124, 137), colon cancer (130), breast cancer (128), hepatocellular carcinoma (HCC) (139, 140), bladder cancer (141) and medulloblastoma (142) (see Table 4 for a complete list of studies which measured exon 9 inclusion in cancer). In addition, a pan-cancer analysis of NUMB exon 9 inclusion utilizing TCGA RNA-sequencing data confirmed these prior studies and also reported upregulation of NUMB exon 9 across breast cancer subtypes as well as in ovarian, prostate, and testicular cancer (Table 4) (Zhang, 2022 #1146). Thus alterations in NUMB exon 9 splicing are a common event across multiple tumor types.

Reduction of total NUMB protein expression has also been reported in human breast, lung, salivary, and colon cancer (111, 143, 144, 145, 146), and associated with deregulation of Notch activity and loss of p53 activity, leading to the early description of NUMB as a tumor suppressor. Biochemical analysis by Misquitta *et al.* demonstrated that exon 9 inclusion correlates with reduced total NUMB protein expression (124, 137). Thus changes in isoform expression may provide an explanation for the tumor associated reduction of total NUMB protein levels through an unknown mechanism.

**Figure 5:**
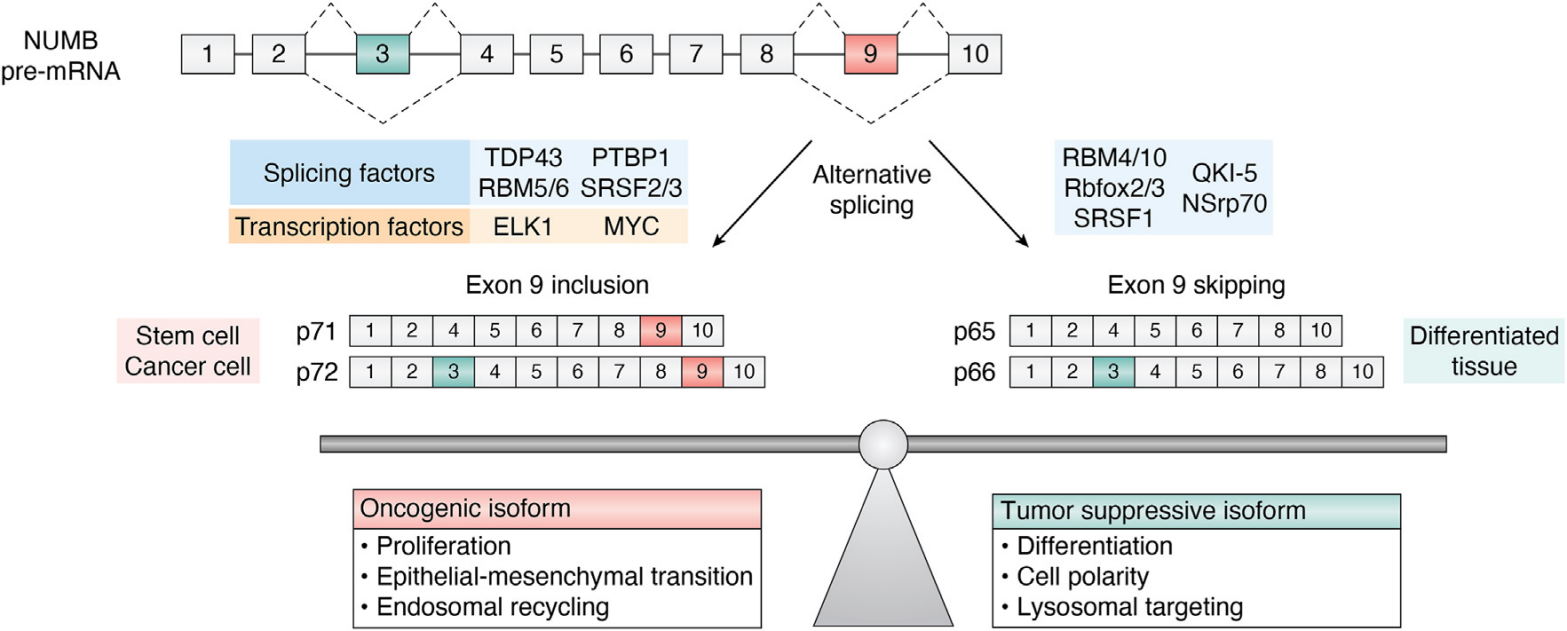
**Regulation of NUMB alternative splicing in cancer progression.** A schematic representation of NUMB gene organization with factors that regulate exon 9 splicing in cancer cells shown below in *blue* and *orange*. The protein products of exon 9 inclusion and exon 9 skipping are depicted showing p72 and p71 dominantly expressed in stem cells and cancer cells, and p66 and p65 expressed in differentiated cells. The dominant functions ascribed to the exon 9 included and skipped isoforms are listed below and described in the text.

Several of the splicing factors demonstrated to regulate NUMB isoform expression during development are themselves misregulated in cancer (Fig. 5). For instance, mutation or altered expression of the RBM family of splicing factors, including RBM5, RBM6 and RBM10, has been described in a number of cancer types (Table 3). RBM5 is downregulated in lung and prostate cancer and is a component of a 17 gene metastasis associated signature (129, 147, 148, 149, 150). In breast cancer, altered expression of RBM5 as well as RBM6 and RBM10 mRNA and protein levels have been reported (151, 152). RBM10 is frequently downregulated in lung, colorectal, pancreatic and endometrial cancers (150, 153, 154, 155, 156) and loss of function mutations have been identified in lung adenocarcinoma (153, 157). RBM family proteins exhibit an antagonist relationship in regulation of NUMB exon 9 splicing in which RBM6 can promote exon 9 inclusion, while RBM10 promotes exon 9 skipping (129). Similarly, RBM10’s effect on NUMB exon 9 splicing was observed in endometrial cancer where it has also been shown that miR-355 can impact overall RBM10 levels (158). Loss of function mutations in RBM10 identified in lung adenocarcinoma (153, 157) disrupt RBM10 function leading to increased exon 9 inclusion, and increased proliferation of the lung adenocarcinoma derived A549 cell line (129, 157).

SRSF3, together with hnRNP family splicing factor TDP43, also regulates cancer-associated NUMB exon 9 splicing (128). Comparison of the alternative splicing patterns in breast cancer subtypes (luminal A, luminal B, Her2-enriched, and TNBC) based on percent-spliced-in (PSI), found a unique splicing profile in triple-negative breast cancer that included NUMB exon 9. Knockdown of either, or both TDP43 and SRSF3 using shRNA in MDA-MB-231 breast cancer cell lines increased NUMB exon 9 skipping and was associated with reduced proliferation (128).

PTBP1, a member of the heterogenous nuclear ribonucleoprotein family is overexpressed in a number of cancers including glioma (159, 160), ovarian cancer (161), breast cancer (162), and colon adenocarcinoma (130), and promotes the expression of several protein isoforms associated with tumorigenesis including NUMB Ex9in. A strong positive correlation between PTBP1 expression and NUMB exon 9 inclusion was shown across 48 human tissues and cell lines (130). In addition, siRNA-mediated knockdown of PTBP1 reduces exon 9 inclusion (113, 130), and overexpression can upregulate exon 9 inclusion (113).

The splicing factor, SRSF1 is overexpressed in several cancers including breast, lung, and colon (163, 164, 165) causing altered splicing of several genes associated with tumorigenesis (166). SRSF1 has been demonstrated by both knockdown and overexpression in A549 lung cancer and HEK293 cells to promote exon9 skipping (113). This observation is at odds with studies showing that overexpression of SRSF1 promotes exon inclusion in genes linked to oncogenic phenotypes including angiogenesis (VEGF), proliferation (Cyclin D1), cellular motility (RAC1 & RON) as well as disrupting tumor suppressor activity (BIN1) (166). To date there have been no correlative analyses between SRSF1 and NUMB transcripts in human tumors. Whether SRSF1 upregulation is associated with decreased Ex9in, as might be predicted from the *in vitro* splicing reporter assays, or is subject to more complex post-translational regulation or context dependent effects as described below remains to be determined (167).

The activity of splicing factors is subject to regulation through post-translational modification from upstream pathways. For example, phosphorylation of the serine/arginine-rich region in SR proteins impacts their localization and activity, thus altering the expression of protein isoforms whose splicing is regulated by those factors (166, 168). SRPK2 is a serine/threonine kinase that regulates the activity and localization of several SR-motif splicing factors including SRSF2 and is frequently overexpressed in cancer (169, 170, 171). Depletion of SRPK2 impairs the invasion and migration of HCT116 colon cancer cells and reduces NUMB exon 9 inclusion (139, 171). NUMB splicing is also regulated by the MAPK/ERK pathway. Inhibition of MEK1/2 reduces NUMB exon 9 inclusion in lung and breast cancer cell lines while, expression of constitutively active MEK1 or V12-HRAS, causes increased exon 9 inclusion (113). This effect of RAS-ERK signaling on NUMB splicing may be mediated in part by the PTBP1 splicing factor since activated RAS signaling increases expression of PTBP1, which in turn promotes NUMB exon 9 inclusion (130). Thus, in addition to mutation or overexpression of splicing factors, activation of oncogenic signaling pathways provides a mechanism to alter NUMB isoform expression.

### How does exon 9 including NUMB isoforms promote tumorigenesis?

Multiple studies addressing NUMB isoform functions support a general model in which increased expression of exon 9 included isoforms contribute to tumor growth and metastasis by antagonizing the tumor suppressive function of the exon 9 skipped NUMB isoforms (Fig. 5). The involvement of NUMB exon 9 splicing in the regulation of Notch signaling, cell proliferation and migration has been investigated in a number of studies, however, as described below, the observed effects of individual isoform expression may be dependent on the specific cellular context and likely the ratio of endogenous isoform expression. For example, in breast cancer MDA-MB-468, and lung cancer A549 cell lines, overexpressing of Ex9sk NUMB isoform decreased colony number and/or suppressed cell growth whereas Ex9in overexpression exerted no change (91, 129). In human breast cancer MDA-MB-231 cells, overexpression of the Ex9sk isoforms decreases cell doubling rate and colony forming ability whereas overexpression of Ex9in isoforms has the opposite effects (128). The differential effect of isoform expression ratio is also supported by Lu *et al.* (139) who compared the effects of NUMB knockdown in hepatocellular carcinoma cells lines expressing different ratios of Ex9in and Ex9sk isoforms. Depletion of NUMB in Huh1 cells (primarily Ex9in expression) resulted in decreased proliferation, while depletion in SK-Hep1 (primarily Ex9sk expression) caused increased proliferation.

In mammalian cells, the p66 isoform of NUMB (Ex9sk) can negatively regulate Notch signaling and target gene expression by promoting lysosomal sorting and degradation of the Notch receptor (64). Isoform-specific rescue of NUMB-depleted A549 cells revealed that while p72 (Ex9in) expression alone had no effect on Notch target gene expression, overexpression of p66 (Ex9sk) inhibited Notch target expression to levels seen in non-targeted control A549 cells (124). Interestingly, co-expression of Ex9sk p66 with Ex9in p72 increased Notch target gene expression. Similarly, in Huh7 cells that express both Ex9in and Ex9sk isoforms, NUMB-depletion caused decreased cell growth. Re-expression of Ex9in NUMB isoform did not affect cell growth while re-expression of an Ex9sk isoform further inhibited cell growth. Together these observations support the notion that in the context of Notch signaling and cell proliferation, NUMB Ex9in isoforms function to antagonize Ex9sk isoforms.

Several studies have modulated the endogenous expression of Ex9in and Ex9sk isoforms by anti-sense oligonucleotides, isoform-specific knockdown, or by genomic deletion of exon 9. Modulation of endogenous exon 9 inclusion in A549 and HeLa cells using antisense oligonucleotides (AON) showed that whereas increased levels of exon 9 inclusion either had no effect on (A549) or increased colony formation respectively, this was markedly decreased by Ex9sk expression (129) Similarly, forcing exon 9 skipping through CRISPR/Cas9 deletion of exon 9 genomic sequence in MDA-MB-468 and MCF7 cells (91), or shRNA-mediated depletion of Ex9in in A549 cells (132) reduced cell growth and/or colony formation. These studies were extended by examining the effects of modulating exon 9 inclusion on tumorigenesis in orthotopic xenograft mouse models. Increased NUMB exon 9 inclusion (by AON) in A549 cells promoted early primary tumor growth compared to controls, whereas tumors with increased Ex9sk expression exhibited slower growth (129). In an orthotopic breast cancer xenograft mouse model CRISPR/Cas9 deletion of exon 9 in MDA-MB-468 cells resulted in the marked reduction of spontaneous lung metastases (91). Similarly, in a xenograft model of pancreatic cancer, CRISPR/Cas9 deletion of exon 9 in AsPC-1 pancreatic cells decreased liver metastases compared to control (172).

The underlying mechanism for NUMB Ex9in isoform effects in cancer cells is in part likely due to alterations in endocytic regulation of multiple membrane receptors. In addition to Notch receptor signaling, isoform-specific roles in endocytic trafficking and activity of the oncogenic receptor tyrosine kinase ALK have been reported (92). In addition, using an unbiased proteomic approach, Zhang *et al.*, found that the removal of NUMB Ex9in isoforms in MDA-MB-468 breast cancer cells resulted in the remodeling of endocytic network proteins, and downregulation surface proteins involved in signaling, migration tumor metastasis (91, 92). Together these studies point to a common mechanism that underlies the cancer-associated functions of Ex9in isoforms and suggest that NUMB isoform expression may bias endocytic trafficking and signaling of multiple cell surface receptors and adhesion molecules.

### Exon 3 isoforms' role in disease

As described above, the exon 3 encoded sequence, located within the PTB domain of NUMB, is important for the interaction between the PTB domain and the E3 ligase MDM2. This interaction between Ex3in NUMB and MDM2 disrupts the ubiquitination of p53 by MDM2, thereby preventing p53 degradation and maintaining p53 tumor suppressor activities. Isoform-specific siRNA knockdown of Ex3in, but not Ex3sk NUMB, in MCF10A cells line (p53 wildtype) strongly reduces p53 levels and leads to persistent DNA damage following cisplatin treatment (18). Stratification of 890 breast cancer patients into Ex3in high (highest quantile 80%) and Ex3in low groups (lowest quantile 20%) showed that Ex3in low group had a higher risk of distant metastasis, especially within the p53 WT group of patients in univariate analysis (18). Thus, loss of Ex3in isoform expression may contribute to cancer progression in specific patient subgroups with wild-type p53 tumors. To date, altered exon 3 splicing has not been identified as a frequent event in cancer. Indeed, analysis of TCGA breast cancer data showed a large gradient of exon 3 inclusion level across tumor samples which was not significantly different from tissue matched normal samples in most cancer types (91). Therefore, levels of exon 3 inclusion or skipping in cancer may be determined by the tissue of origin rather than oncogenic signaling or alterations in the activity of the splicing machinery.

The cellular response to stress includes changes in NUMB exon 3 splicing. For instance, trophic factor withdrawal (TFW) induces elevated Ex3sk NUMB levels in several neuronal tissues. Using an Ex3sk-specific antibody to measure NUMB Ex3sk protein in primary mouse cultured hippocampal neurons, TFW was shown to elevate NUMB Ex3sk splice variants within 6 h of treatment (173). This supports earlier studies by Kyriazis *et al.*, in rat PC12 neuro-endocrine tumor cells, where stress induced by TFW resulted in a rapid and reversible increase in Ex3sk NUMB mRNA, with a corresponding decrease in Ex3in transcripts (78, 174). In contrast, ethanol-induced gastric damage in murine stomach causes elevated Ex3in (both p66 and p72) mRNA (175). Skipping of NUMB exon3 also appears to correlate with Alzheimer’s disease (AD) progression. Ex3sk-specific immunofluorescent labeling of NUMB in the parietal cortex of Alzheimer’s disease (AD) patients and age matched controls indicated significantly elevated expression in the AD samples (173). Ex3sk NUMB protein was also selectively elevated in lysates prepared from the cortex of 12 month old triple transgenic AD model mice (173). The accumulation of extracellular amyloid β-peptide (Aβ) plaques in the brain is one of the pathological hallmarks of AD (176) and aberrant processing of the APP to generate Aβ is central to the pathogenesis of AD. As described above, Ex3sk NUMB isoform expression can prevent post-endocytic degradation of APP and promote its processing to Aβ (78), suggesting a mechanistic link between increased expression of NUMB Ex3sk isoform expression and Aβ accumulation in AD.

## Conclusion and perspectives

Vertebrate NUMB protein isoforms were identified over 2 decades ago (10), yet studies exploring NUMB protein function in endocytosis, cell migration and adhesion, development and disease have generally not taken into account the potential for distinct isoform activity in their design and interpretation. While it is increasingly clear that a core conserved function of NUMB proteins from *C elegans* to humans is to regulate recycling of membrane receptors, understanding the complex functions of vertebrate NUMB requires consideration of individual isoform effects on membrane and protein interactions, trafficking pathways, and the ratio of isoform expression in particular cell types.

The conserved function of NUMB Ex9sk isoform to inhibit the recycling of membrane receptors during development is essential to limit available Notch signaling to regulate cell fate and limit growth factor and adhesive signaling. The detailed mechanisms that underlie this activity remain unknown, as is the importance of binding to adaptins and endocytic proteins for its function in recycling. Despite this, evidence suggests that in vertebrates, the additional NUMB isoforms modulate this function of NUMB. In particular, the expression of exon 9 included isoforms in stem cells and progenitors as well as in disease states such as cancer can suppress the inhibitory activity of other NUMB isoforms. While most of the evidence to date supports this type of antagonistic relationship, whether exon 9-containing isoforms also harbor gain of function activity remains unknown. An additional detailed comparison of the biochemical and structural properties of the NUMB isoforms is likely to provide a mechanistic understanding of how exon 9 sequences confer unique activity to individual isoforms.

Splicing of NUMB exon 9 is likely part of a broader program of coordinately spliced genes that contribute to programs of differentiation during development, malignant transformation, and tumor progression. Changes in the ratio of NUMB isoform expression control cargo selection and the fate of membrane receptors within the endocytic pathways. In addition, exon 9 splicing may influence NUMB transcription or translation giving rise to a reduction in total NUMB protein expression. Alternate splicing of exon 3 likely provides another mechanism to fine-tune the function of NUMB by influencing membrane localization and cargo selection. How the ratio of exon 3 containing isoforms is regulated, and whether the patterns of exon 3 and exon 9 splicing are mechanistically linked to favor expression of specific isoforms is an area of ongoing investigation.

Signal-mediated regulation of NUMB isoform expression allows fine-tuning of cargo selection and receptor recycling and degradative fates during development. This mechanism is co-opted in disease states such as cancer through oncogenic signaling, or aberrant activity of splicing factors to alter NUMB isoform expression. As such, the mechanisms involved in regulating NUMB splicing may provide a window into potential strategies to restore NUMB isoform expression for therapeutic benefit in diseases such as cancer.

## Data availability

All supporting data arew provided within the manuscript, supplementary data and supplementary tables.
